# A study on music therapy aimed at psychological trauma recovery for bereaved families driven by artificial intelligence

**DOI:** 10.3389/fpsyg.2024.1436324

**Published:** 2024-10-08

**Authors:** Zixi Wang, Xin Guan, Enhui Li, Bo Dong

**Affiliations:** ^1^College of Music and Dance, Guangzhou University, Guangzhou, China; ^2^Guangzhou Xinhua University, Dongguan, China

**Keywords:** music therapy, bereaved families, psychological trauma repair, Long Short-Term Memory network, effect evaluation

## Abstract

**Introduction:**

This study explores the effectiveness of music therapy in repairing psychological trauma in bereaved families, aiming to provide a comprehensive understanding of its potential therapeutic impact. It begins with an analysis of the current situation faced by bereaved families, identifying the psychological challenges they experience.

**Methods:**

The research design included the recruitment of participants from bereaved families, who were then divided into an experimental group and a control group. An optimized Long Short-Term Memory (LSTM) network model was constructed to analyze music therapy data, tailored specifically to capture the nuances of this therapeutic process. The experimental procedure detailed the specific operations involved in the music therapy sessions and established a clear comparison framework between the two groups.

**Results:**

The performance of the proposed LSTM model demonstrated high accuracy (0.85), precision (0.86), recall (0.84), and *F*_1_-score (0.85), indicating its effectiveness in predicting treatment outcomes. When compared to the Self-Reporting Inventory-90 (SCL-90) scale, the model captured the trend of treatment effects with a high level of accuracy and reliability. Notably, participants numbered 2, 6, and 8 in the experimental group showed substantial improvement rates of 67.21%, 71.45%, and 75.67%, respectively, in their psychological health issues.

**Discussion:**

The comparative analysis between the experimental and control groups confirmed that the music therapy approach, as guided by the proposed LSTM model, led to a more significant improvement in psychological health issues for bereaved families. This suggests that the model offers a promising avenue for enhancing the effectiveness of music therapy in this context.

## 1 Introduction

Bereaved families refer to those families who lost their only child for various reasons during the implementation of China's one-child policy. The implementation of this policy has resulted in many families in China being able to have only one child for a long time. Therefore, the loss of this unique child has been an extremely devastating and unbearable blow to the family (Myers-Coffman, 2024). Bereaved families face unprecedented psychological trauma and difficulties, which often last for many years or even a lifetime, greatly affecting the mental health of family members. Firstly, the loss of the only child is an indescribable pain for parents. In traditional Chinese culture, children are the main reliance and spiritual support for parents in their old age (Ghetti et al., 2023). However, once the only child is lost, parents not only lose the process of raising children but also lose the continuity and inheritance of the family. This sense of loss and helplessness often leads parents to long-term immersion in extreme sadness, and even severe depression and anxiety. Secondly, the marital relationship of bereaved families also faces enormous tests and challenges. Under the one-child policy, many families place all their love and hope on their only child. Once the child is lost, the relationship between spouses often falls into a state of mutual blame and estrangement (Gillespie et al., 2024). The emotions and trust between spouses may also be severely damaged, leading to the breakdown of family relationships and the failure of marriages. In addition, the elders and other family members of bereaved families also find it difficult to escape from psychological distress and negative effects. For the elders of bereaved families, losing their only grandson or granddaughter means losing the continuation of family affection and hope, often leading to deep anxiety and loneliness. Other family members, such as siblings, relatives, and friends, also often feel the pain and pressure of bereaved families, which often affects the harmony and stability of the entire family (Moriconi and Cantero-García, 2022).

The psychological trauma and challenges faced by families who have lost their only child are not limited to individual levels but have wide-ranging and profound impacts at the family and societal levels. Firstly, the family structure and function of such families suffer severe disruptions. In traditional family structures, children bear the responsibility of family continuity and inheritance. Losing the only child means the interruption of family continuity and inheritance, leading to irreversible changes in the family structure. This change results in the loss and breakdown of family functions, leaving these families lacking sufficient support and coping abilities when facing challenges and pressure. Secondly, social support and recognition for families who have lost their only child also face significant difficulties. In traditional Chinese culture, the family serves as the basic unit of social life. However, the emergence of families who have lost their only child challenges this traditional notion, resulting in insufficient social recognition and support for such families. On the contrary, they often face criticism, isolation, and even social marginalization. This societal pressure and rejection exacerbate the psychological trauma and suffering of members of these families, making them even more isolated and helpless. Furthermore, the psychological trauma experienced by families who have lost their only child presents challenges to social governance and services. Given that the psychological health issues of these families often persist for a long time and are difficult to treat, they require professional psychological support and therapy services. However, the current psychological health service system for families who have lost their only child is not yet well-developed, facing issues such as insufficient service resources and a lack of professional talents, making it difficult for members of these families to receive timely and effective help and support. This further intensifies the psychological plight of these families and presents new challenges and tasks for social governance and services.

Against the backdrop of significant psychological trauma and challenges faced by bereaved families, it becomes crucial to seek effective methods for psychological trauma recovery. While traditional psychological therapy methods can alleviate the psychological distress of bereaved family members to some extent, they often fail to achieve the desired results due to their limitations and shortcomings (Cheatley et al., 2022). Therefore, it is particularly important to explore a more effective and personalized approach to psychological trauma recovery by combining emerging artificial intelligence (AI) technology with traditional music therapy methods. AI, as an emerging technology, has shown tremendous potential and prospects in the medical field (Myers-Coffman et al., 2020). AI technology can not only help doctors improve the accuracy and efficiency of diagnosis and treatment but also provide patients with more personalized and precise medical services. In the field of psychotherapy, AI technology is also widely used in emotion recognition, psychological assessment, personalized treatment plan design, and other aspects, bringing new possibilities and opportunities for psychotherapy. Meanwhile, music therapy, as a traditional psychological treatment method, has been proven to have significant effects on psychological trauma recovery (Tuomi et al., 2021). Music, as a non-verbal art form, possesses unique functions of emotional expression and emotion regulation, directly influencing people's emotions and psychological states. In music therapy, therapists guide patients to express and release emotions and promote psychological recovery and self-awareness through carefully selected and designed music. Therefore, music therapy is considered a safe, effective, and popular psychological treatment method, widely used in the treatment of various mental health issues.

This study aims to explore AI-driven music therapy methods for psychological trauma recovery in bereaved families, providing more effective and personalized psychological support and assistance. By combining emerging AI technology with traditional music therapy methods, this study aims to provide a novel psychological trauma recovery solution for bereaved family members, helping them rebuild confidence and hope, and reintegrate into social life. The innovation of this study lies in the integration of emerging AI technology and traditional music therapy methods to explore a new treatment approach for psychological trauma recovery in bereaved families. An emotion analysis model tailored to the characteristics of music therapy data is being constructed based on optimized deep learning algorithms to more accurately analyze emotional changes in bereaved family members during music therapy sessions. This innovative approach is expected to enhance the effectiveness and personalization of music therapy, providing better psychological support and assistance to bereaved family members and offering new insights and methods for the development of the field of psychotherapy. This study comprises six sections: Introduction, Literature Review, Research Methodology, Results Analysis, Discussion, and Conclusion. Section 2 reviews the current state of research in relevant fields. The Section 3 introduces the research design, participant recruitment, and the construction of the emotion analysis model based on optimized deep learning algorithms. The Section 4 details the changes in the psychological states of bereaved family participants and examines the effects of AI-driven music therapy on psychological trauma recovery. It also analyzes the outcomes of psychological trauma under various individual differences. In Section 5, the significance of the research findings is explained, differences between AI-driven music therapy and traditional music therapy are discussed, and limitations of the study and future directions for improvement are analyzed. Finally, in Section 6, the research findings are summarized, the potential role of AI-driven music therapy in psychological trauma recovery in bereaved families is emphasized, and suggestions for future research and clinical practice are proposed. This study also contributes to exploring the application of AI technology in the field of psychotherapy, providing new insights and methods for the future development of psychotherapy methods, enhancing understanding of psychological trauma in bereaved families, and promoting social attention and care for bereaved family members.

## 2 Literature review

In recent years, with social changes and diversification of family structures, research on the psychological trauma of bereaved families has gradually attracted attention from academia and society. Many studies have investigated the psychological status and living conditions of bereaved family members in depth, exploring the impact of bereavement on the mental health of family members, and proposing some psychological support and intervention measures (Loewy et al., 2021). For example, Cuervo-Suárez et al. (2022) conducted a study investigating the psychological health status of bereaved parents and its influencing factors. The study used a combined qualitative and quantitative approach, employing surveys and in-depth interviews, and showed that bereaved parents generally experienced severe psychological distress and negative emotions such as depression and anxiety. The study results showed that the psychological health of bereaved parents was influenced by various factors such as social support, family support, and individual factors, and required more care and support from society and family. Additionally, some research indicates that the psychological health issues of bereaved parents also affect marital relationships and family harmony. Love et al. (2022) proposed that the emotional support and communication level between bereaved parents were closely related to the quality of marital relationships, which in turn directly affected the harmony and stability of the entire family. Therefore, providing psychological support and intervention measures for bereaved families not only helped improve individual mental health but also helped maintain family relationships and promoted family harmony.

Music therapy, as a non-pharmacological, non-verbal psychological treatment method, has attracted attention in the field of psychological trauma recovery. Many studies have shown that music therapy plays a positive role in psychological trauma recovery, helping to facilitate emotional expression, emotional regulation, and psychological recovery (Apuke et al., 2023). Lu (2024) investigated the application effects of music therapy in patients with Post-Traumatic Stress Disorder (PTSD) after trauma. They used a randomized controlled trial design, dividing PTSD patients into a music therapy group and a conventional treatment group, and compared the treatment effects of the two groups of patients. The results showed that patients receiving music therapy had significant improvements in anxiety, depression, and sleep quality. Compared to the conventional treatment group, the treatment effect of the music therapy group was more significant and lasting. In addition, a meta-analysis study conducted by Ha (2022) comprehensively analyzed the effects of music therapy in psychological trauma recovery. The study included data from multiple relevant studies and evaluated and analyzed the overall effect of music therapy. The results showed that music therapy had a significant effect in psychological trauma recovery, not only alleviating patients' psychological symptoms but also improving their mental health and quality of life. Moreover, other studies have indicated that music therapy has a certain effect in various age groups and different populations. For example, research on music therapy for children and adolescents has found that music therapy can help them release emotions, enhance self-esteem and confidence, and promote psychological recovery and social skills development (Bensimon, 2020).

The application of AI technology in the field of psychological trauma recovery has gradually gained attention and has achieved some positive research results. AI technology provides new possibilities and methods for psychological therapy through emotion recognition, personalized treatment plan design, and other methods (Kammin et al., 2024). Liu et al. (2022) explored the application effects of emotion recognition technology in psychological trauma recovery. Using deep learning algorithms, they conducted real-time recognition and analysis of the emotional states of trauma patients and adjusted treatment plans based on the recognition results. The results showed that personalized treatment plans based on emotion recognition technology could better meet the psychological needs of patients, thereby improving treatment effectiveness and satisfaction. Additionally, research suggests that AI technology can also assist therapists in psychological assessment and intervention plan design. For example, Baglione et al. (2021) used machine learning algorithms to predict individual characteristics and treatment responses of trauma patients and provided therapists with personalized intervention plans. The results showed that personalized intervention plans based on machine learning algorithms could significantly improve treatment effectiveness and patient satisfaction. Furthermore, research indicates that virtual reality technology has also achieved some positive effects in psychological trauma recovery. Virtual reality technology combines AI technology and psychological therapy methods to provide patients with an immersive treatment experience, which helps promote emotional expression and emotion regulation (De Luca et al., 2022).

Current research on psychological trauma recovery in bereaved families has achieved some positive results. Firstly, in-depth investigations into the psychological health of bereaved families provide a theoretical basis for psychological therapy. Additionally, music therapy, as a non-pharmacological, non-verbal psychological treatment method, has shown effective roles in psychological trauma recovery. Moreover, the application of AI technology, such as emotion recognition technology and machine learning algorithms, provides new possibilities for psychological therapy and more means for designing personalized treatment plans and psychological assessments. The exploration of virtual reality technology also provides patients with immersive treatment experiences, which help promote emotional expression and emotion regulation. However, these studies still have some limitations, including insufficiently rigorous research designs and the need for further improvement in technological applications. Therefore, this study aims to combine AI technology and music therapy methods to explore a novel treatment approach for psychological trauma recovery in bereaved families, with the goal of providing more effective and personalized psychological support and assistance to bereaved families.

## 3 Music therapy research methodology for psychological trauma recovery in bereaved families

### 3.1 Research design and participant recruitment

This study selects a city in inland China as the research area and employs a randomized controlled trial design to ensure the scientific validity and credibility of the experimental results. Participant recruitment is based on the following criteria.

Firstly, selection criteria for participants include: Bereaved family members. Participants must be members of bereaved families, i.e., families who have lost a child. Psychological trauma: Participants exhibit symptoms of psychological trauma, such as depression, anxiety, etc., which need to be clinically diagnosed and confirmed.

Secondly, exclusion criteria for participants include: Severe psychological disorders: Participants with severe mental illnesses or psychological disorders, such as schizophrenia, are excluded from the study. Inability to accept music therapy: Participants who have strong aversions to music therapy or are unable to undergo treatment are also excluded.

During the participant recruitment process, research information is widely disseminated through various channels such as community notices, hospital announcements, internet platforms, etc., inviting eligible bereaved family members to participate. Simultaneously, researchers communicate face-to-face with potential participants, providing detailed explanations of the research content, risks, benefits, and clarifying the voluntary nature of participation, confidentiality, and the right to withdraw at any time.

**Figure 1:**
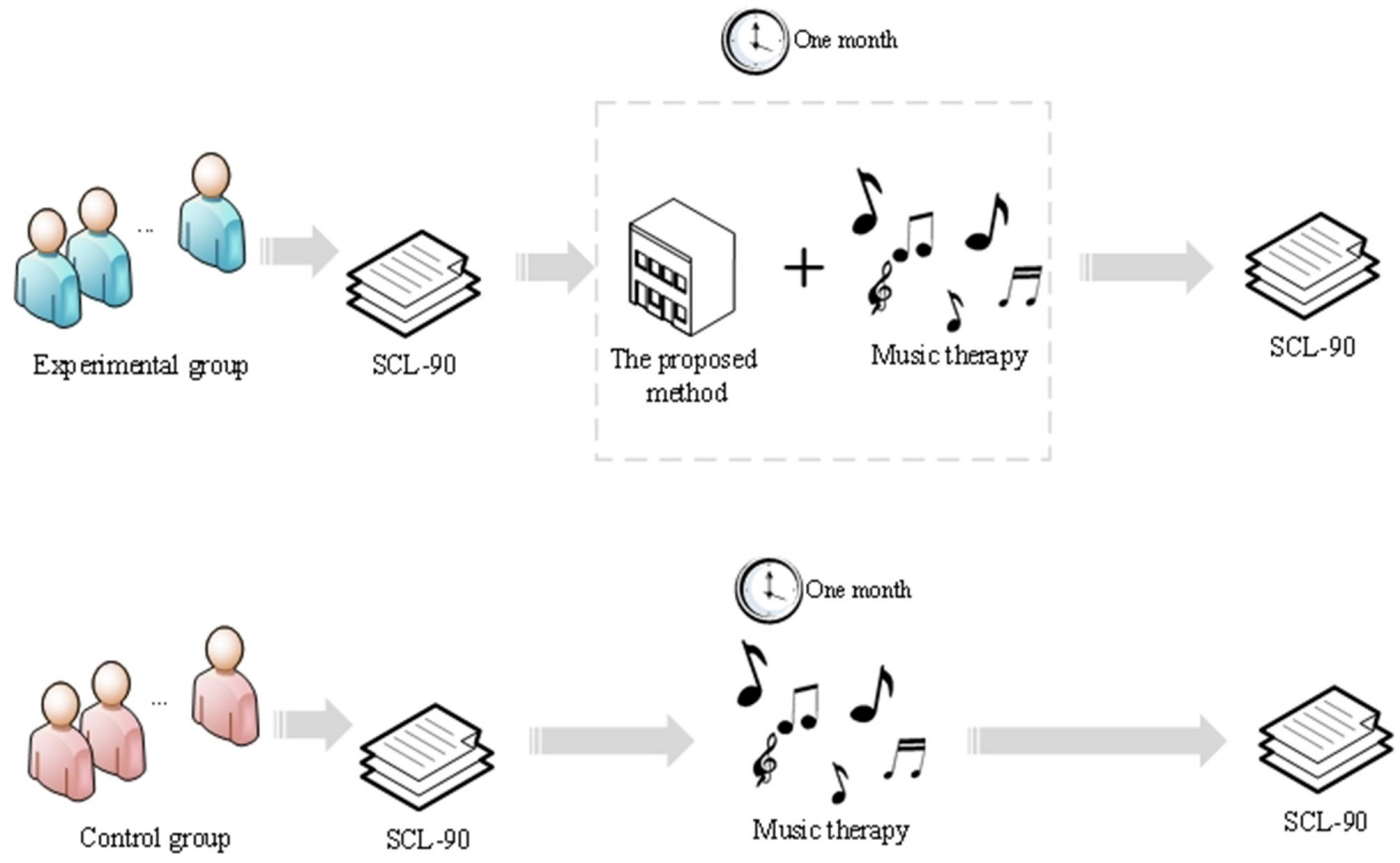
Research design.

A total of 480 participants are ultimately recruited. After determining the participants, they are randomly assigned to the experimental group and the control group, with 240 individuals in each group. The experimental group receives music therapy driven by an optimized Long Short-Term Memory (LSTM) algorithm, while the control group receives traditional music therapy, as shown in Figure 1.

### 3.2 Construction of deep learning model

To address the characteristics of music therapy data, an optimized LSTM algorithm is adopted for model construction. LSTM is a variant of Recurrent Neural Network (RNN) that can effectively handle sequential data and capture long-term dependencies in long sequential data, making it suitable for processing time-series data such as music therapy process data. In this study, the LSTM model is optimized to better adapt to the characteristics of music therapy data and improve the model's prediction and analysis capabilities.

**Figure 2:**
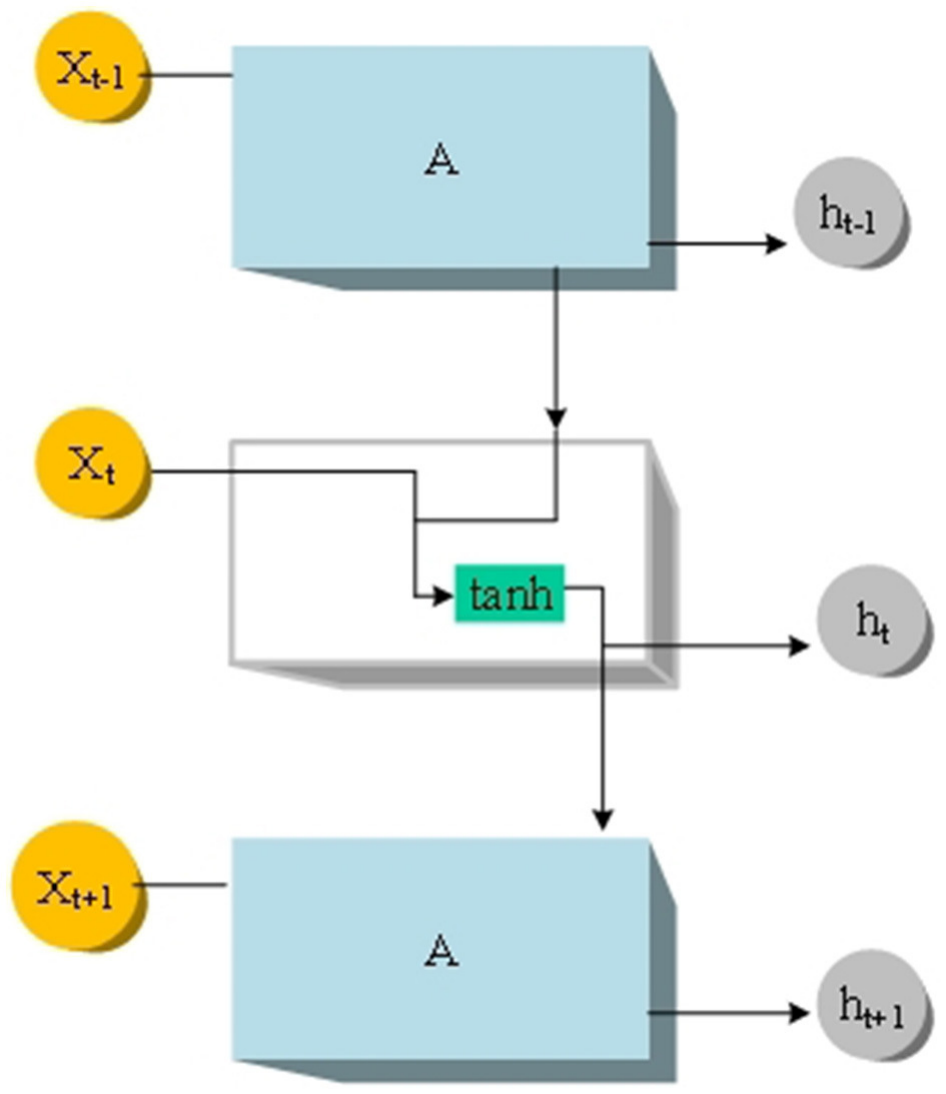
LSTM model structure.

The LSTM network is a special type of RNN with gating mechanisms designed to handle and learn long-term dependencies in sequential data. Compared to traditional RNNs, LSTM introduces three key gate structures: the input gate, forget gate, and output gate, along with a cell state. These gate structures enable the LSTM network to selectively control the flow of information when processing sequential data, thereby more effectively capturing long-term dependencies within sequences. Firstly, the input gate determines which information from the current time step's input should be passed to the cell state. It achieves this by using a sigmoid activation function to control the relevance of each input, ensuring that only relevant information is passed to the cell state. Secondly, the forget gate determines which information in the cell state should be discarded or ignored. It accomplishes this by using a sigmoid activation function and element-wise multiplication to decide which information to forget, thereby preventing the cell state from being overly populated or containing irrelevant information. Lastly, the output gate determines which information from the current time step's cell state should be outputted to the next state and prediction. The output gate controls the information output from the cell state using a sigmoid activation function and another tanh activation function to map the information from the cell state into the range of output values. The cell state is the core of the LSTM network, allowing information to persist over long periods and mitigating the vanishing or exploding gradient problems present in traditional RNN. Through the cell state, the LSTM network can effectively transmit and capture information when processing long sequential data, thereby enhancing the network's performance and generalization ability. LSTM, as a type of RNN with gating mechanisms, better handles long-term dependencies in sequential data through the design of gate structures and cell states. It exhibits strong memory and generalization capabilities, leading to its widespread application and research across various sequential modeling tasks. The structure of the LSTM model is depicted in Figure 2 (Reid and Miño, 2021).

The basic computational formulas for LSTM are shown in Equations 1–5:


(1) $$\begin{array}{r} \begin{array}{c} i_{t}=\sigma \left(W_{xi}x_{t}+W_{hi}h_{t-1}+W_{ci}c_{t-1}+b_{i}\right) \end{array} \end{array}$$

(2) $$\begin{array}{r} f_{t}=\sigma \left(W_{xf}x_{t}+W_{hf}h_{t-1}+W_{cf}c_{t-1}+b_{f}\right) \end{array}$$

(3) $$\begin{array}{r} c_{t}=f_{t}⊙c_{t-1}+i_{t}⊙tanh\left(W_{xc}x_{t}+W_{hc}h_{t-1}+b_{c}\;\right) \end{array}$$

(4) $$\begin{array}{r} o_{t}=\sigma \left(W_{xo}x_{t}+W_{ho}h_{t-1}+W_{co}c_{t}+b_{o}\right) \end{array}$$

(5) $$\begin{array}{r} h_{t}\&=o_{t}⊙tanh\left(c_{t}\;\right) \end{array}$$

In Equations 1–5, *x*_*t*_ represents the input at the *t*-th time step of the input sequence; *h*_*t*_ is the hidden state at the *t*-th time step of the output sequence; *c*_*t*_ is the cell state; σ denotes the sigmoid function; ⊙ indicates element-wise multiplication; *i*_*t*_, *f*_*t*_, and *o*_*t*_, respectively, represent the outputs of the input gate, forget gate, and output gate; *W* and *b* are the weight parameters and bias terms of the model.

The LSTM model is optimized to improve its effectiveness in psychological trauma repair, considering the characteristics of music therapy data. The optimization focuses on several aspects:

1. Sequence length handling

Data generated during music therapy often have varying sequence lengths, presenting a challenge for input and processing in the model. To address variable-length sequence data, a dynamic time matching technique is employed. Specifically, a Bidirectional RNN (Bi-RNN) is used to dynamically capture the features of sequence data, and the sequences of different lengths are transformed into fixed-length inputs through weighted averaging (McFerran, 2020). Assuming the input sequence is *X* = {*x*_1_, *x*_2_, …, *x*_*T*_}, where *T* is the length of the sequence, the Bi-RNN model yields bidirectional hidden representations of the sequence, denoted as *H* = {*h*_1_, *h*_2_, …, *h*_*T*_}. The weighted averaging method aggregates these hidden representations, as shown in Equation 6:


(6) $$\begin{array}{l} \bar{h}=\overset{T}{\underset{t=1}{\sum }}\alpha_{t}h_{t} \end{array}$$

In Equation 6, α_*t*_ represents the dynamically calculated weighting coefficient based on the importance of hidden representations.

2. Feature extraction

To address the characteristics of music therapy data, an effective feature extraction method is designed to extract key information from the music therapy process, which serves as input to the LSTM model. This process can be divided into several steps:

Audio signal preprocessing: Firstly, the audio signal undergoes preprocessing, including framing and time-frequency feature extraction. Assuming the audio signal sequence is *x* = {*x*_1_, *x*_2_, …, *x*_*T*_}, where *T* is the length of the sequence. The audio signal is framed into multiple windows, and each window's audio signal can be represented as a vector *x*, where *t* is the time step. Then, time-frequency features are extracted from the audio signal of each window using Short-Time Fourier Transform (STFT) to convert the time-domain signal into a time-frequency domain representation, obtaining the time-frequency feature matrix *X*. The calculation formula for STFT is given in Equation 7:


(7) $$\begin{array}{l} X\left(m,\omega \right)=\overset{N-1}{\underset{n=0}{\sum }}x\left[n\right]\cdot w\left[n-m\right]\cdot e^{-j\omega n} \end{array}$$

In Equation 7, the input signal is denoted as (*t*), the window length is *N*, and the window function is *w*(*t*). *X*(*m*, ω) represents the STFT result at time index *m*m and frequency index ωω, *x*[*n*] is a discrete time-domain sample of the input signal, *w*[*n*] is the window function, and *e*^−*jωn*^ is the rotation factor.

Extracting audio features using convolutional neural network (CNN): Next, a CNN is employed to extract advanced representations of audio features. CNNs can effectively capture local features of audio signals and extract audio features crucial for the task. A CNN model is constructed using convolutional layers, pooling layers, etc., with the time-frequency feature matrix *X* as the input, resulting in the advanced representation *F*_*audio*_ of audio features.

Integration of psychological state data: Finally, the advanced representation *F*_*audio*_ extracted by the CNN is fused with the psychological state data from the music therapy process to obtain a comprehensive feature representation as input to the LSTM model. Assuming the psychological state data sequence is *y* = {*y*_1_, *y*_2_, …, *y*_*T*_}, it can be represented as a feature vector *y*_t_. Then, the advanced representation of audio features *F*_*audio*_ and the feature vector *y*_t_ of psychological state data are concatenated or added together to obtain the final feature representation *F*_*final*_, as shown in Equation (8):


(8) $$\begin{array}{l} {\text{F}}_{final\;}=concatenate\left({\text{F}}_{audio\;},{\text{y}}_{t}\right) \end{array}$$

3. Parameter tuning

By conducting repeated experiments and parameter tuning, various parameters of the LSTM model have been optimized, including learning rate, hidden layer size, and number of iterations, aiming to improve the convergence speed and prediction accuracy of the model. Parameter tuning is a crucial part of the training process of deep learning models, directly impacting the performance and generalization ability of the model. The following common parameter tuning methods are employed.

Random search is a simple yet effective method for parameter tuning, which involves randomly selecting parameter combinations within a predefined parameter space and training and evaluating each combination. Unlike traditional grid search methods, random search does not exhaustively search the parameter space but randomly samples within it, allowing for exploration of a wider range of parameter combinations in a shorter time. This random parameter selection approach imbues random search with a degree of exploration, aiding in the discovery of superior parameter settings. Due to its flexibility, random search can adapt to the requirements of different tasks and models, as it is not constrained by the size of the parameter space. Additionally, parallelizing random search implementation is relatively straightforward, enabling simultaneous training and evaluation of multiple parameter combinations across multiple computing resources, thereby enhancing tuning efficiency. Overall, random search, as a simple yet powerful parameter tuning method, has found wide application in the training process of deep learning models. Its characteristic of random sampling enables it to search for better-performing parameter settings in a shorter time, providing crucial support for model performance improvement.

Grid search is a common method for parameter tuning that employs an exhaustive search strategy by traversing all possible parameter combinations within a specified parameter grid. In grid search, candidate values for each parameter are predefined, and these values are combined to form a parameter grid. The grid search algorithm then trains and evaluates each parameter combination within the grid to determine the optimal combination. While the search space in grid search is typically limited, especially when there are many parameters and candidate values, the search process can become time-consuming. However, the advantage of grid search lies in its ability to ensure exhaustive search of all possible combinations within the parameter space. Since grid search traverses every corner of the parameter space, it can identify the globally optimal parameter settings without missing any possible combinations. One of the main advantages of grid search is its simple and intuitive implementation, making it one of the commonly used tuning tools in many machine learning tasks. By exhaustively searching within a limited parameter grid, the grid search algorithm can provide comprehensive coverage of the parameter space, helping us find the optimal parameter combination to optimize model performance and generalization ability.

Cross-validation is a commonly used method for evaluating model generalization performance. It involves partitioning the dataset into training and validation sets and repeatedly training and evaluating the model to reduce the risk of overfitting. During the parameter tuning process, cross-validation is used to evaluate the performance of each parameter combination to select the optimal parameter settings. Through cross-validation, the model's performance on unseen data can be more accurately assessed, thereby improving the model's generalization ability and preventing overfitting to the training data.

To further enhance the predictive performance of the model, this study introduces Bayesian Optimization as the hyperparameter optimization method. Bayesian Optimization is an advanced optimization technique that constructs a probabilistic model based on prior knowledge to iteratively explore the parameter space in search of the global optimum. Specifically, Bayesian Optimization builds a surrogate model (often a Gaussian Process or tree-based model) in each iteration to approximate the objective function, such as the validation error of the model. By using acquisition functions like Expected Improvement (EI), Bayesian Optimization focuses the search on high-potential regions, thus more efficiently identifying the optimal parameter combination. Compared to traditional random search and grid search methods, Bayesian Optimization significantly reduces the number of trials required for parameter tuning, as it leverages information from previous evaluations to guide the selection of the next candidate parameters. This technique not only converges to the global optimal parameters more quickly with fewer computational resources but also manages complex dependencies between parameters, thereby improving the model's predictive performance and generalization ability. In this study, Bayesian Optimization will be used to adjust key hyperparameters of the LSTM model, such as learning rate, hidden layer size, and number of iterations. Through these optimizations, the study aims to further improve the model's accuracy, making its predictions in psychological trauma recovery more precise.

In the process of parameter tuning, the parameter combination that performs best on the validation set is selected based on the model's performance on the validation set, and this combination is used for the final training and testing of the model. This ensures that the model has good generalization ability on unseen data, thus better predicting and analyzing the effects of music therapy on psychological trauma repair in bereaved families. The music dataset chosen in this study is the Giant Musical Instrument Digital Interface (MIDI)-Piano dataset, released by ByteDance, which contains a large amount of classical piano music MIDI data covering various genres and styles of music. The final parameter combination determined in this study is: a learning rate of 0.00005, a hidden layer size of 256, and 150 iterations.

To further prevent overfitting and enhance the model's generalization ability, this study introduces more robust regularization methods on top of the optimized LSTM model. These methods include L_2_ regularization (weight decay) and Dropout techniques, both of which are widely used in deep learning to effectively improve the model's robustness and generalization performance.

L_2_ regularization works by adding a penalty term to the loss function that is proportional to the square of the model weights. This regularization method effectively limits the size of the model weights, thereby reducing the risk of overfitting. Specifically, the effect of L_2_ regularization on the loss function is expressed by the formula in Equation 9:


(9) $$\begin{array}{l} L=L_{\text{original}}+\lambda \overset{n}{\underset{i=1}{\sum }}w_{i}^{2} \end{array}$$

In Equation 9, *L*_original_ represents the original loss function, λ is the regularization strength parameter, and *w*_*i*_ are the model's weight parameters. By adjusting the value of λ, one can control the strength of the regularization, thereby balancing the model's complexity and its fitting capability.

Dropout is a commonly used regularization technique in deep learning. It reduces co-adaptation among neurons by randomly dropping out (i.e., setting the activation values to zero) a portion of neurons during each training iteration. This approach helps the model learn more robust feature representations across different training epochs, thus improving its generalization ability. Specifically, during training, neurons are randomly selected and dropped with a certain probability *p* (typically between 0.2 and 0.5), while during testing, all neurons are retained and the outputs are scaled accordingly, as shown in Equation 10:


(10) $$h_{i}=\{\begin{array}{c} 0\;with\;probability\;p\\ \frac{h_{i}}{1-p}\;with\;probability\;1-p \end{array}$$

In Equation 10, *h*_*i*_ represents the activation value of a neuron, and *p* is the dropout probability. Dropout prevents model overfitting and improves performance on the test set by reducing dependencies between neurons.

By combining L_2_ regularization and Dropout techniques, the optimized LSTM model demonstrates enhanced robustness in handling complex patterns in music therapy data. It is expected to show improved performance on test data, exhibiting better generalization capability.

### 3.3 Music therapy process and control group setup

The music therapy process in this study aims to explore the impact of music on psychological trauma repair in bereaved families and adopts a control group design to validate the treatment effect. During the music therapy process, participants in the treatment group receive specially designed music therapy, while participants in the control group receive conventional psychological support or other non-music interventions. The duration of the experiment for both groups is 1 month.

The selection of music is based on classical piano pieces, covering a variety of styles and emotional characteristics of music to ensure the diversity and specificity of the treatment. Music environment creation: during the music therapy process, a quiet and comfortable environment is created for participants to maximize their listening and experiencing of music. Therapy process supervision: the therapy process is supervised and guided by professionally trained music therapists to ensure that participants can fully engage in the music experience. Emotional expression and relaxation: participants are guided to express their inner emotions through music, release emotional stress, and relax their minds and bodies with the accompaniment of music, promoting psychological recovery. Participants in the control group receive similar music therapy to the treatment group.

To conduct a more in-depth analysis of individual differences among participants in the music therapy process, this study introduces an analysis dimension for individual differences within the experimental group of 240 individuals. Participants are first grouped based on key demographic factors (such as age, gender, and economic background) and psychological status (initial SCL-90 scores). These groups is used to explore variations in music therapy outcomes among different characteristic groups.

Specifically, the study employs hierarchical regression analysis to assess the impact of various factors on treatment efficacy. Additionally, it examines the interaction effects among different characteristic groups to identify factors that may lead to significant differences in treatment outcomes. Through this design, the study aims to better understand how individual differences affect the efficacy of music therapy and provide insights for future personalized treatment plans.

### 3.4 Ethical approval

This study was approved by the Academic Ethics Review Committee of Guangzhou University, China, on February 12, 2024. Our study did not involve animal or human clinical trials and was not unethical. In accordance with the ethical principles outlined in the Declaration of Helsinki, all participants provided informed consent before participating in the study. The anonymity and confidentiality of the participant guaranteed, and participation was completely voluntary. At the same time, researchers communicate face-to-face with potential participants, providing detailed explanations of the research content, risks, benefits, and clarifying the voluntary nature of participation, confidentiality, and the right to withdraw at any time.

## 4 Analysis of research results on music therapy for psychological trauma repair in bereaved families

### 4.1 Model performance evaluation

The performance of the proposed optimized LSTM algorithm in this study is evaluated and compared with four other common deep learning models, including Multilayer Perceptron (MLP), CNN, LSTM Networks, and Gate Recurrent Unit Networks (GRU). The results are shown in Table 1.

**Table 1:** Comparison of model performance.

**Model**	**Accuracy**	**Precision**	**Recall**	***F*_1_-score**
The model proposed in this study	0.88	0.89	0.87	0.88
MLP	0.79	0.81	0.77	0.79
CNN	0.82	0.83	0.81	0.82
LSTM	0.84	0.85	0.83	0.84
GRU	0.81	0.82	0.8	0.81

**Figure 3:**
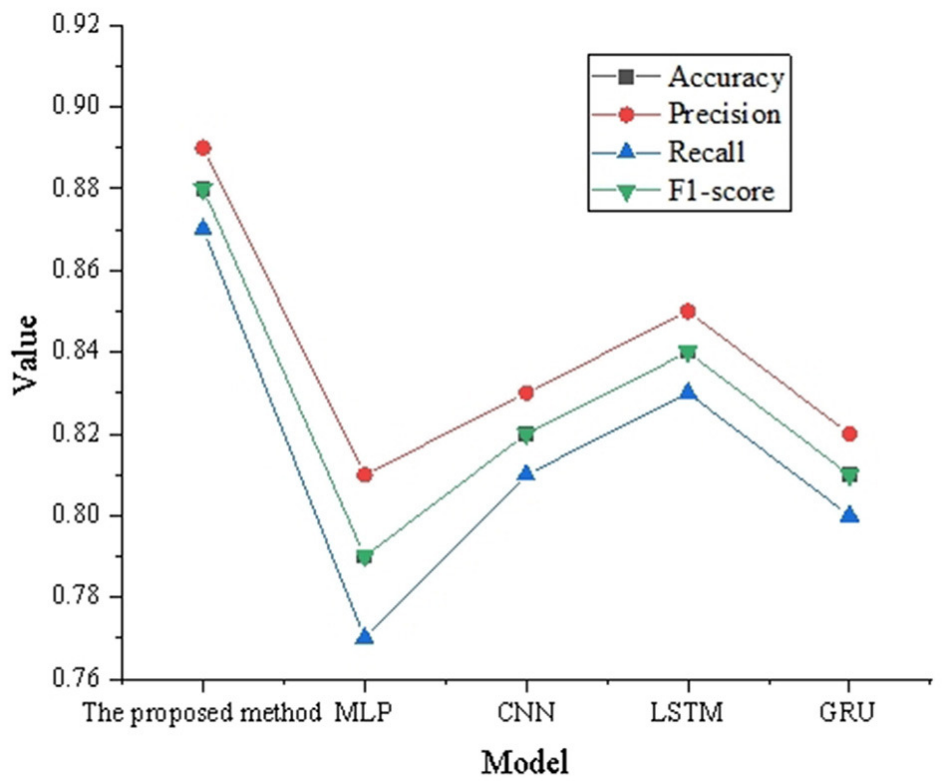
Comparison of model performance results.

To provide a more intuitive representation, a graph is plotted based on the content of Table 1, as shown in Figure 3.

Table 1 and Figure 3 show that the model proposed in this study demonstrates superior performance across accuracy, precision, recall, and *F*_1_-score, achieving values of 0.88, 0.89, 0.87, and 0.88, respectively. In comparison, the performance of MLP and GRU is slightly inferior, with accuracy and *F*_1_-score slightly lower than other models, at 0.79 and 0.81, respectively. Meanwhile, CNN and LSTM show performance similar to the model proposed in this study but with slightly lower recall rates. Overall, the LSTM algorithm proposed in this study demonstrates significant advantages in predicting the effect of music therapy on the psychological trauma recovery of bereaved families, exhibiting better predictive and generalization abilities.

### 4.2 Analysis of differences in results between the experimental and control groups

**Figure 4:**
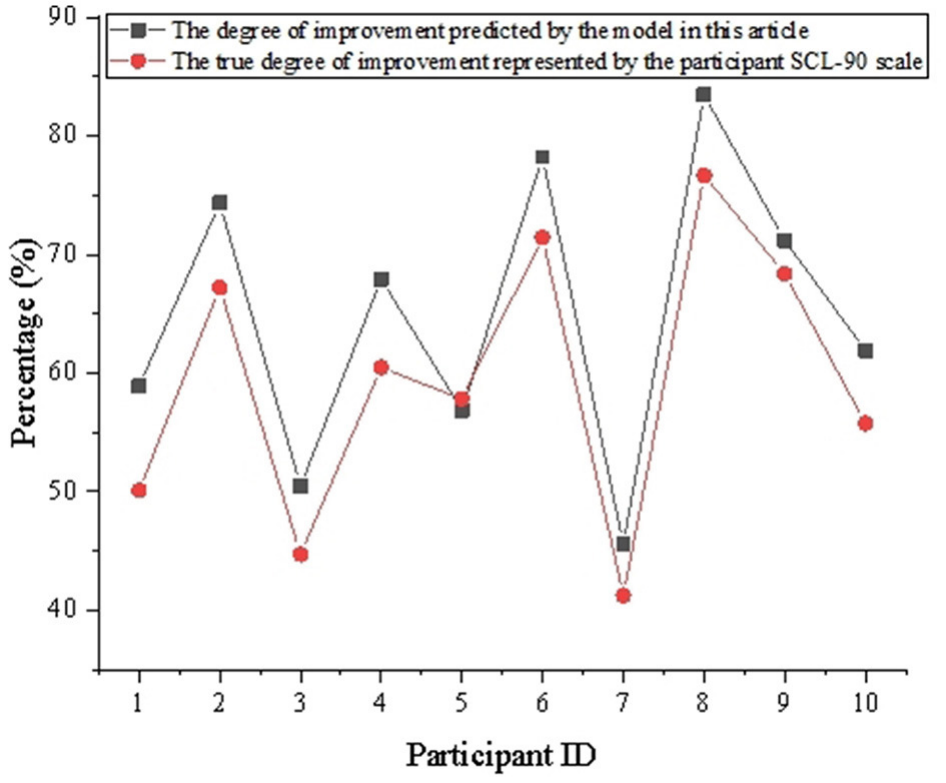
Evaluation of model predictive effectiveness.

This study selects the Self-reporting Inventory 90 (SCL-90) scale to evaluate the therapeutic effects. Initially, a comparative analysis is conducted between the treatment effects predicted by the model for the experimental group and the results of the SCL-90 scale completed by the experimental subjects at the end of the experiment. Due to the consideration of space, only the treatment data of 10 participants in the experimental group, out of 240 individuals, are listed in this study. To simplify the analysis process and compare the degree of improvement, the factors of the SCL-90 are merged, and the results are shown in Figure 4.

Figure 4 demonstrates that the therapeutic effects predicted by the model in this study have a certain consistency with the improvement levels characterized by the SCL-90 scale reported by the participants. For example, for participant number 2, the improvement degree predicted by the model proposed in this study is 74.35%, whereas the actual improvement degree as represented by the SCL-90 scale is 67.21%. This trend is also visible in other participants, indicating a correlation between the predicted improvement level and the actual characterized improvement level. This suggests that the model in this study has a certain accuracy and reliability in predicting the effects of music therapy. Although the predictions are not entirely accurate, the model can capture the general trend of the therapeutic effects. The predictive ability and guiding therapeutic capacity of the model have significant implications for music therapy.

**Figure 5:**
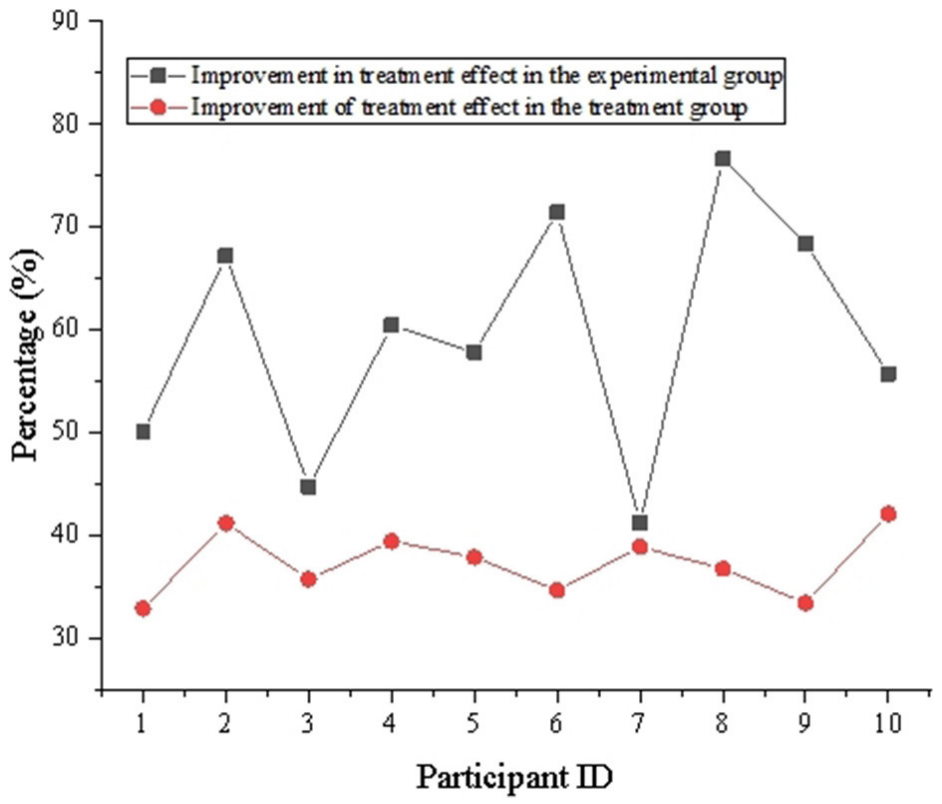
Comparison of treatment effects.

Furthermore, by comparing the improvement data of psychological problems before and after treatment for 10 participants each in the experimental and control groups, the greater improvement in psychological health issues in the experimental group can be more intuitively illustrated, indirectly indicating the feasibility of the model proposed in this study, as shown in Figure 5.

Figure 5 demonstrates that the experimental group performs better in terms of treatment effects. For example, the improvement in treatment effects for participants numbered 2, 6, and 8 in the experimental group reached 67.21, 71.45, and 76.67%, respectively. In contrast, these participants show significantly lower improvement in the control group. Conversely, some participants in the control group (such as participants numbered 1, 3, and 6) have lower treatment effect improvements, even falling below the average level of the experimental group. This data indicates that using the model proposed in this study for music therapy in the experimental group has significant advantages and can more effectively improve participants' mental health issues.

### 4.3 Individual difference analysis

Descriptive statistics are used to analyze the treatment effects for different characteristic groups. Table 2 shows the treatment effect improvement percentages (%) across various age groups, genders, and initial SCL-90 score categories.

**Table 2:** Descriptive results of treatment effects for different characteristic groups.

**Characteristics**	**Grouping**	**Number of samples**	**Average improvement (%)**	**Standard deviation (%)**
Age	< 40 years old	80	72.34	8.45
	40–60 years old	100	65.89	9.12
	>60 years old	60	58.67	10.23
Gender	Male	120	64.75	9.57
	Female	120	70.23	8.76
Economic background	Low income	70	61.2	9.89
	Middle income	100	67.89	8.34
	High income	70	73.45	7.76
Initial SCL-90 score	High (>160)	80	75.12	7.89
	Middle (100–160)	100	67.45	8.97
	Low (< 100)	60	62.89	9.34

Table 2 shows that different characteristic groups exhibit significant differences in psychological health improvement following music therapy. For instance, younger participants (< 40 years) show a higher average improvement compared to older participants (>60 years), who have relatively lower improvement. Female participants have a higher average improvement compared to males, and participants with a favorable economic background show more pronounced treatment effects. Participants with higher initial SCL-90 scores experience the greatest improvement, which may indicate a high responsiveness to the treatment in these groups.

To quantify the impact of various factors on treatment outcomes, a hierarchical regression analysis was conducted to assess the independent contributions of age, gender, economic background, and initial SCL-90 scores to treatment effects. In the regression analysis, one group is selected as the baseline (e.g., males or low-income) and other groups (e.g., females or high-income) are compared relative to the baseline group. This approach simplifies the model, avoids multicollinearity issues, and more clearly demonstrates the differences in treatment effects across different characteristic groups. Therefore, the regression analysis results present coefficients relative to the baseline group rather than listing all variables. The results are shown in Table 3.

**Table 3:** Hierarchical regression analysis results.

**Variables**	**Regression coefficient (B)**	**Standard error (SE)**	***t*-value**	***p*-value**
Age (< 40 years)	0.35	0.08	4.38	0.0001
Age (40–60 years)	0.22	0.07	3.14	0.002
Gender (female)	0.28	0.06	4.67	0.0001
Economic background (high income)	0.4	0.07	5.71	0.00001
Initial SCL-90 score (high)	0.45	0.09	5	0.00001

Based on the hierarchical regression analysis results in Table 3, age, gender, economic background, and initial SCL-90 scores all significantly affect treatment outcomes. Notably, initial psychological state has the greatest impact on treatment effects, with a regression coefficient of 0.45, indicating that individuals with poorer initial psychological states benefit more from the treatment. Economic background also has a significant effect on treatment outcomes, with higher income participants showing greater improvement (*p* < 0.00001). These results highlight the importance of individual characteristics in music therapy and provide crucial references for future personalized treatment plans.

## 5 Discussion

This study first evaluates the performance of the proposed optimized LSTM algorithm and compares it with four other common deep learning models. The results show that the proposed model achieves the best performance in accuracy, precision, recall, and *F*_1_-score. In contrast, the performance of other models, particularly multilayer perceptrons and gated recurrent units, is somewhat inferior, with slightly lower accuracy and F_1_-scores. This indicates that the proposed optimized LSTM algorithm offers significant advantages in predicting the effectiveness of music therapy for psychological trauma recovery in bereaved families, demonstrating superior predictive and generalization capabilities. The model's advantages primarily stem from the use of advanced optimization methods such as Bayesian optimization and regularization techniques, along with finely tuned parameters. Bayesian optimization efficiently explores the parameter space, allowing the model to converge quickly to a global optimum and further enhance predictive accuracy. L_2_ regularization and Dropout techniques effectively prevent overfitting and improve the model's generalization ability, resulting in excellent performance across accuracy, precision, recall, and *F*_1_-score metrics. These optimization measures collectively contribute to the model's significant advantages in predicting music therapy outcomes. In the analysis of differences between the experimental and control groups, the experimental group using the proposed model shows better performance, further validating the accuracy and reliability of the model in predicting music therapy effects. The model's superiority is not only due to its comprehensive understanding and learning ability for music therapy data but also its capacity to capture the complex mechanisms of psychological trauma recovery in bereaved families, providing a powerful tool for therapists. Additionally, through the analysis of individual differences in the experimental group, this study further reveals the significant impact of demographic factors (such as age, gender, economic background) and psychological state (such as initial SCL-90 scores) on treatment outcomes. Hierarchical regression analysis finds that younger participants, females, those with better economic backgrounds, and individuals with poorer initial psychological states show greater improvement in treatment. These findings provide important references for future personalized music therapy plans and suggest that individual differences should be thoroughly considered when developing treatment plans.

## 6 Conclusion

This study aims to explore the effect of music therapy on the psychological trauma repair of families who have lost their only child, and proposes an optimized LSTM algorithm based on deep learning models to predict and guide the music therapy process. Through comparative analysis between the experimental and control groups, as well as comparison between model predictions and actual treatment outcomes, the following conclusions are drawn in this study. The predictive ability and guiding role of the model help therapists better understand and guide patients' treatment processes, thereby achieving better treatment outcomes. The experimental results show that patients in the experimental group who undergo music therapy using the model in this study perform better in improving their mental health issues, further validating the effectiveness and guiding role of the model. Individuals with poorer initial psychological states benefit more from the treatment. The impact of economic background on treatment outcomes is also significant, with high-income participants showing greater improvement (*p* < 0.00001). Although there is still some prediction error, the model can capture the general trend of treatment effects with a certain level of accuracy and reliability. However, this study has some limitations. First, the sample size is relatively small, which may affect the stability and generalizability of the experimental results. Although the study involves 480 participants, providing a certain degree of representativeness within the research scope, the focus on a city in inland China results in a culturally and socio-economically homogeneous sample, potentially limiting the generalizability of the findings. Lastly, the study only reports on treatment effects 1 month after intervention and lacks long-term follow-up data. Future research should consider expanding the sample size, including participants from diverse cultural and socio-economic backgrounds, optimizing the model algorithms, and incorporating more psychological theories. Long-term follow-up studies should be designed to track participants' psychological state changes over extended periods to assess the long-term effects and sustainability of music therapy, providing more comprehensive treatment outcome information and ultimately enhancing the model's predictive capability and practical applicability.

## Data Availability

The original contributions presented in the study are included in the article/supplementary material, further inquiries can be directed to the corresponding authors.
